# Spectrum of operative childhood intra-articular shoulder pathology

**DOI:** 10.1007/s11832-014-0598-8

**Published:** 2014-06-18

**Authors:** Eric W. Edmonds, Joanna H. Roocroft, Shital N. Parikh

**Affiliations:** 1 Department of Orthopedic Surgery, University of California San Diego, 3030 Children’s Way, Suite 410, San Diego, CA 92123 USA; 2 Division of Orthopaedic Surgery, Rady Children’s Hospital and Health Center, San Diego, CA USA; 3 Division of Orthopaedic Surgery, Cincinnati Children’s Hospital Medical Center, Cincinnati, OH USA

**Keywords:** Shoulder pathology, Arthroscopy, Pediatric, Children

## Abstract

**Purpose:**

With increased sports participation and medical community awareness, there appears to be an increase in pediatric musculoskeletal injuries. Our purpose was to identify the intra-articular injury pattern seen within the pediatric shoulder.

**Methods:**

A retrospective review was performed at two tertiary-care children’s hospitals between 2008 and 2011 on all patients who underwent magnetic resonance imaging (MRI) and subsequent shoulder arthroscopy. Exclusion criteria included: girls >14 years old and boys >16 years old. Demographics, MRI and arthroscopic findings were recorded. Labral pathology was grouped: Zone I (Bankart lesions, 3–6 o’clock for right shoulder), Zone II (posterior labral lesions, 6–11 o’clock), Zone III (SLAP lesions, 11–1 o’clock), and Zone IV (anatomic variants, 1–3 o’clock).

**Results:**

One hundred and fifteen children met criteria, mean age 14.4 years (range 8–16). There were 24 girls and 91 boys, with 70 right shoulders. Of 108 children, labral pathology involved: 72 Zone I (16 isolated anterior), 56 Zone II (15 isolated posterior), 38 Zone III (four isolated superior), and three had an isolated Buford complex. Seventy had more than one labral zone injured, and 31 (30 %) had more than two zones injured. Non-labral pathology included partial rotator cuff tears and humeral avulsions of the glenohumeral ligament.

**Conclusion:**

With 94 % of intra-articular pathology being labral tears, the distribution of proportion in children differs from adults; moreover, 23 % involved only the posterior or posterosuperior labrum. Treating surgeons should be prepared to find anterior tears extending beyond the zone of a classic Bankart lesion and an association with C rotator cuff tears.

## Introduction

Child and adolescent participation in organized athletics has become exceedingly popular, with evidence of high intensity play and minimal periods of rest [1]. An estimated 7 million high school students are participating in sports annually, and although the knee is most commonly injured, nearly 11 % of all injuries occurred at the shoulder [2].

There have also been reports of shoulder injuries in the pre-teenage population, particularly related to instability, labral pathology and shoulder impingement [3–5]. Most children sustain an injury to the developing bone and growth plate rather than the soft-tissues of the glenohumeral joint as in the skeletally mature population [6]. To the best of our knowledge, no epidemiologic studies exist focusing on operative intra-articular shoulder pathology in children. Most shoulder studies that discuss children are confounded by wide age ranges, including late adulthood [7].

Despite an increase in sports participation and awareness on behalf of the medical community of pediatric musculoskeletal injuries, the spectrum of intra-articular shoulder pathology in this skeletally immature population has not been well described. The purpose of this study was to report on the spectrum of operative pediatric intra-articular shoulder injuries.

## Methods

After obtaining Institutional Review Board approval, a retrospective cross-sectional study was performed at two tertiary care children’s hospitals in two distinct geographic locations of the United States. From 2008 through 2011, all patients who underwent shoulder arthroscopy were identified. Reasons for arthroscopic intervention included: recurrent instability (traumatic or multi-directional instability), or persistent symptoms despite conservative management with magnetic resonance (MR) findings of intra-articular pathology. Exclusion criteria included: girls >14 years old, and boys >16 years old to produce a cohort of children, rather than young adults based on expected closure of the proximal humeral physis.

Demographic and injury characteristics, including age, gender, laterality, date of injury, date of imaging study, date of surgery, and sport played at time of injury, were recorded. MR and arthroscopic findings were recorded to include the diagnosis by radiologist or orthopedic surgeon, respectively. MR findings were included only if that data contained extra-articular pathology not identifiable via arthroscopy. If labral pathology was identified, then the extent and location of the tear was evaluated.

**Fig. 1:**
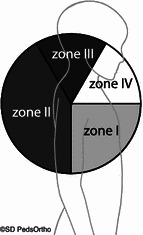
Labral tears were grouped into zones based on the location of the injury

Labral pathologies identified at surgery were grouped into zones based on a clock-face system that we developed, and noted as if appearing at a right shoulder (Fig. 1): Zone I (Bankart lesions) were 3–6 o’clock, Zone II (posterior labral lesions) were 6–11 o’clock, Zone III (superior labrum anterior posterior [SLAP] lesions) were 11–1 o’clock, and Zone IV (anatomic variants) were 1–3 o’clock.

## Results

This study included 115 children (24 girls, 91 boys) with a mean age of 14.4 years (range 8–16 years). Sixty-one percent of the injuries involved the right shoulder. Shoulder injury in boys occurred most commonly during football (51 %) and baseball (31 %); however, at least one injury occurred during lacrosse, volleyball, fighting, go-kart driving/dirt bike, wrestling, soccer, waterpolo, swimming, basketball, hockey, and weightlifting. Girls were injured primarily by basketball (38 %), followed by softball (12.5 %), waterpolo (8 %), volleyball (8 %), gymnastics (8 %), dance (8 %), and swimming (8 %). Many girls (9.5 %) were not injured during a sport, but rather a fall or pop with trauma not otherwise specified.

**Fig. 2:**
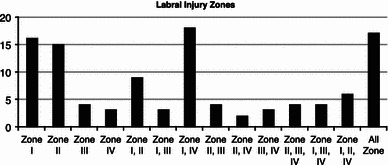
Histogram demonstrating the location of labral pathology in the child shoulder

Of the 108 children with labral pathology, 72 involved Zone I with 16 isolated anterior, 56 involved Zone II with 15 isolated posterior, 38 involved Zone III with four isolated superior, and three had an isolated Buford complex at final diagnosis. Injuries were isolated to a single zone of injury in 38 (34 %) children. Seventy (65 %) children were injured in more than one labral zone, and 31 (29 %) were injured in more than two labral zones (Fig. 2). Posterior and posterosuperior pathology was recorded in 25 children, including isolated Zone II, Zone II and III, Zone II and IV, as well Zones II, III and IV (with IV really just being a Buford lesion).

Pathology in Zone I included: five anterior labral periosteum sleeve avulsions (ALPSA), ten glenolabral articular disruptions (GLAD), and seven bony Bankart lesions. There were seven children with isolated non-labral intra-articular pathology, including five with an isolated pathologic capacious capsule. There were 32 children with labral tears who had associated non-labral pathology, including: 29 rotator cuff tendon injuries (partial articular supraspinatus tendon avulsions—PASTA), two humeral avulsions of the glenohumeral ligament (HAGL), and one greater tuberosity fracture.

## Discussion

Intra-articular pathology seen in the adult shoulder includes, but is not limited to: rotator cuff disease, biceps tendon pathology, labral pathology, osteoarthritis and instability; and, according to our results, the spectrum of pathology in children appears to differ from adults, with the primary intra-articular pathology (93 %) being labral pathology. Nearly a quarter (23 %) involved the posterior labrum with or without the superior labrum, and about two-thirds involved at least two zones of injury. Moreover, almost one-third of our cohort (28 %) had partial rotator cuff tears, but there were no complete tears during the collection period.

The present study of children undergoing shoulder arthroscopy compares well with another multicenter instability registry (which only included teenagers) that demonstrated 83 % anterior instability and 10 % posterior instability, although isolated posterior labral pathology in our study was slightly more common, at 16 % [8]. Thus, our findings may be generalizable to intra-articular shoulder pathology at other institutions; particularly considering that our two institutions are geographically dissimilar.

A study by Owens and colleagues evaluating shoulder instability at a military academy found that the 1-year incidence of instability was 2.8 % [9]. In this older population of college-aged students, they found a slightly higher proportion of anterior dislocation (80 %) and a lower proportion of posterior instability (10 %), compared to 67 and 20 %, respectively, found in our study. Although instability was not directly measured, zones of injury were measured, with 18 % of tears being purely anterior (Zone I and IV), 15 % purely posterior, and 6 % isolated SLAP tears. The remaining tears included those children with multiple zones of injury, either representing greater degrees of pathology or multi-directional pathology. Our higher prevalence of posterior pathology is concerning, because previous work on posterior pathology in adults highlighted an almost 18 % increased risk for recurrent posterior instability within the first year of treatment, being even greater in patients under 40 years of age [10].

Moreover, this same study evaluating young adult military recruits with a mean age of 20 years had a slightly smaller prevalence of women (14 %) with intra-articular pathology compared to the girls in our study (21 %). A recent study on an adolescent population evaluating PASTA injuries had 28 % girls in their cohort [4]. It seems that these two studies on girls demonstrate a higher prevalence of shoulder injury than the study on young adult women. Perhaps age is a protective factor for the female population, accounting for the higher proportion of girls seen in both our study and other adolescent studies compared to reports of similar pathology in young adult women.

Partial rotator cuff tears have been well studied in adolescents by previous authors, and it seems the consensus is that this pathology can often occur in association with other intra-articular lesions, especially superior labral tears [4, 7]. The mechanism for this pathology is primarily participation in overhead sports, especially internal impingement; but, injury to the rotator cuff can occur from instability as well [4, 7].

The only epidemiology studies performed on the child shoulder are radiographic studies evaluating the accuracy of the diagnostic test [11, 12]. In a diagnostic study on the presence of upper extremity pathology in children, Davis confirmed that overuse injuries involving the growth plates were more common than previously known [11]. Moreover, rotator cuff tears and labral injuries were present in this young cohort, but prevalence was not discussed.

With the current understanding of intra-articular pathology in the skeletally immature, decisions can be made regarding treatment. Previous authors have highlighted the need to consider skeletal maturity when treating shoulder pathology in children, because of their unique anatomy and activity level [1]. On the one hand, arguments are made favoring an emphasis on prevention of injury through family education followed by as-needed surgery if an injury does occur. And on the other hand, there is an argument encouraging nonsurgical treatment in this otherwise adaptable population. Taylor and associates demonstrate that although adolescent patients may be adaptable to injury, they are also at higher risk for recurrent pathology due to their high activity level [1]. Therefore, we support the first argument that emphasizes education and prevention, but requires surgery to repair these tears to reduce the risk of recurrent injury and progression of pathologic changes.

The limitation to this study is the retrospective design, which allows for the possibility that patients were excluded due to criteria of incomplete charts or that data was not discovered in review. Furthermore, intra-articular shoulder injuries are not common in the skeletally immature, and therefore, there is a low rate of surgical intervention in children—as seen in past study on children [13]. Moreover, the presence of anatomic lesions does not necessarily correlate with clinical symptoms in children, and this study did not evaluate the clinical relevance of the identified pathology.

Despite its limitations, this study demonstrates a contrast to the expected adult intra-articular pathology, with a higher rate of labral pathology and partial rotator cuff tears seen in the pediatric shoulder. Perhaps this can be accounted for by the etiology, wherein children tend to have traumatic injuries and adults are prone to non-traumatic pathology. Although associated pathology was identified in 30 % of the children, 94 % of the skeletally immature cohort had primary labral pathology. Additionally, children may have a higher incidence of posterior labrum involvement than even the young adult population, and girls appear to have greater risk for injury than skeletally mature females. If intra-articular pathology is suspected in children, then the treating surgeon should investigate the presence of glenoid labral tears that extend beyond a single zone of injury.
